# Detection of *invA* gene of *Salmonella* from milkfish (*Chanos chanos*) at Sidoarjo wet fish market, Indonesia, using polymerase chain reaction technique

**DOI:** 10.14202/vetworld.2019.170-175

**Published:** 2019-01-29

**Authors:** Sheila Marty Yanestria, Reina Puspita Rahmaniar, Freshinta Jellia Wibisono, Mustofa Helmi Effendi

**Affiliations:** 1 Department of Veterinary Public Health, Faculty of Veterinary Medicine, Wijaya Kusuma University, Surabaya, Indonesia; 2 Department of Microbiology, Faculty of Veterinary Medicine, Wijaya Kusuma University, Surabaya, Indonesia; 3 Department of Veterinary Public Health, Faculty of Veterinary Medicine, Airlangga University, Surabaya, Indonesia

**Keywords:** human health, *invA* gene, milkfish, polymerase chain reaction, *Salmonella*

## Abstract

**Aim:**

The study aimed to detect the *invA* gene in *Salmonella* isolated from milkfish in the Sidoarjo wet fish market.

**Materials and Methods:**

A total of 84 samples were prepared in enrichment media and isolated on the surface of *Salmonella* Shigella Agar. *Salmonella* growth produces transparent colonies with blackish color in the middle due to H_2_S gas formation. Samples were identified as *Salmonella* based on macroscopic colony morphology. Presumptive *Salmonella* sp. was put on Bismuth Sulfite Agar media. *Salmonella* was determined based on the results of the biochemical test that has been carried out using Microbact identification kits from negative gram staining.

**Results:**

The results of this study indicate that 32 of 84 samples (38.09%) were *Salmonella* bacteria. Furthermore, the *invA* gene detection was carried out using the polymerase chain reaction technique. Electrophoresis results showed four positive samples contained *invA* gene with a length of 284 bp.

**Conclusion:**

Results in this study indicate that contamination of milkfish with *Salmonella* needs strict hygienic measures to prevent their transmission to human.

## Introduction

Indonesia is an agricultural country that has a large enough fishery potential. The amount of capture fisheries production in 2014 was 6.20 tons, an increase of 0.34 million tons from 2013 which was only 5.86 million tons. The amount of fish production from the aquaculture sector has also increased from year to year [1]. Some fish products contaminated with bacteria can cause a disease called foodborne disease. Foodborne disease or also called foodborne diarrheal disease is a zoonotic disease and is found throughout the world. Foodborne diarrheal disease is a disease transmitted by animal’s carriers that are healthy to humans through food contamination [2].

One of the bacteria that cause foodborne disease is *Salmonella* and is called salmonellosis. This disease is endemic in almost all major cities in Indonesia, and the incidence of typhoid fever caused by *Salmonella typhi* continues to increase with the incidence of 350-810 cases per 100,000 population with a mortality rate of 2%. Typhoid fever is found throughout the year [3]. There are several transmission routes for salmonellosis, but the majority of human infections are derived from the consumption of contaminated foods, especially those of animal origin. Most cases of salmonellosis in fish start from consuming raw fish meat without first processing and cross-contamination [4]. The US Food Drug Administration reports data that *Salmonella* is the most common contaminant bacteria found in fish and fish products [5]. Established conventional methods to detect and identify *Salmonella* are time-consuming and include selective enrichment and plating followed by biochemical tests [6].

*Salmonella* detection can be done in various ways, one of which is using the polymerase chain reaction (PCR) technique. This technique can be used on very complex sample substances such as food and is able to work quickly and specifically. Due to the advantages, PCR is suitable to detect the presence of *Salmonella*. Detection of *Salmonella* using PCR can be done in a short time with high accuracy so that appropriate handling can be done on food [7]. The *invA* gene from *Salmonella* contains unique DNA sequences and is proven to be a PCR target gene suitable for *Salmonella* detection [8].

In this research, samples from milkfish were tested for isolation of *Salmonella*, by culturing and biochemical method, and then, they were confirmed by *invA* gene-specific PCR methods.

## Materials and Methods

### Ethical approval

Fresh milkfish were used in this study; hence, ethical approval was not necessary. Milkfish samples were collected from Sidoarjo wet fish market.

### *Salmonella* culture method

Milkfish samples are cut with a sterilized knife. Grind the meat and digestive tract of the fish in a mortar with a stemper, then take 1 g and put it in 5 ml of selenite broth, and incubate it for 24 h at 37°C.

Bacterial isolation is carried out by taking bacterial suspensions with inoculating loop which is first sterilized using Bunsen fire. The suspension is then implanted in *Salmonella* Shigella Agar (SSA) which has been labeled according to the sample using the streak method to obtain a separate colony which is then incubated for 24 h in a temperature of 35-37°C. The growth of *Salmonella* produces transparent or colorless colonies with blackish color in the middle due to H_2_S gas formation [9]. Presumptive *Salmonella* is put on Bismuth Sulfite Agar (BSA) media. Bismuth sulfite is a selective medium for *Salmonella* isolation in the laboratory. BSA is a modification of Wilson and Blair Formula. Typhoid-causing organisms grow well in this medium, with the characteristics of black colonies. Gram-positive and other coliform bacteria are inhibited. These media are generally used for the detection of *Salmonella* species.

### Microscopic examination and biochemical test

Positive samples on BSA media were then given Gram staining to determine the type of Gram bacteria that grew. Positive results on Gram examination show that bacteria in the form of long and medium rods, red, Gram-negative bacteria spread perfectly without forming long chains or groups [10]. After Gram staining, the bacteria were tested with biochemical tests [11]. The *Salmonella* was determined based on the results of the biochemical test that has been carried out using Microbact identification kits from OXOID.

### DNA extraction

The initial step of PCR is DNA extraction of bacterial culture in *Salmonella* Shigella media to be extracted by boiling method. The boiling method was carried out for 10 min and centrifuged for 5 min at a speed of 6000 rpm. The supernatant is used for PCR amplification.

### PCR amplification

Detection of *Salmonella* invasive encoding gene was carried out using the PCR test. The reagents for PCR amplification consisted of 2.5 µl DNA template, 5 units of GoTaq DNA polymerase, 1 × GoTaq PCR reaction buffer (containing 1.5 mM MgCl2), 0.2 mM PCR nucleotide mix, and 0.6 μM DNA primers with a final volume of 50 μl. Specific primers used to detect *Salmonella* are primary forward (*invA*) GTG AAA TTA TCG CCA CGT TCG GGC AA and primer reverse TCA TCG CAC CGT CAA AGG AAC C [12].

The PCR reagent mixture was then put in a thermocycler with an initial incubation of 94°C for 1 min, followed by 35 cycles consisting of 94°C for denaturation for 1 min, annealing at 58.3°C for 30 s, and 72°C elongation for 30 s followed by the final extension at 72°C for 7 min. Each of the 3 µl of amplification products was mixed with 3 µl of loading solution until well mixed.

### Electrophoresis of PCR products

The results of PCR product amplification were electrophoresis in 1.5% agarose gel and stained with ethidium bromide. Markers are also inserted into the agarose gel well to determine the DNA size of the PCR product; then, electrophoresis is run for 40 min with a constant voltage of 100 v. Electrophoresis results were observed under ultraviolet light. The results obtained are DNA band patterns (DNA bands) which show different numbers and patterns. 100 bp DNA ladder was used as a marker.

### Sensitivity and specificity of PCR [13]

PCR assay was evaluated in various concentration of DNA and various concentration of viable *Salmonella* Typhimurium for analyzing sensitivity of PCR . For sensitivity test based on DNA concentration, DNA extract was sequentially diluted ten-folds and based on the viable bacterial count, *S*. Typhimurium was sequentially diluted from 10^9^ to 1 colony-forming unit/mL, and DNA was extracted from each dilution. DNA extracted from *Staphylococcus aureus* was used as negative control, in each PCR run. The PCR products were visualized by electrophoresis and observed as described above. The minimum concentration of DNA giving a positive signal was recorded. The specificity of PCR was evaluated by comparing with six different *Salmonella* serovars and cross-tested with 5 non-*Salmonella* isolates, i.e., *Escherichia coli, Listeria monocytogenes, Proteus mirabilis, S. aureus*, and *Vibrio parahaemolyticus* [13].

## Results and Discussion

### Results of *Salmonella* culture and identification

**Figure-1:**
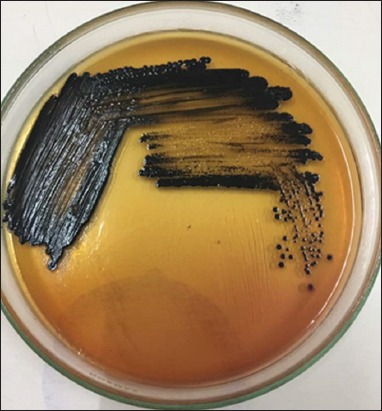
Result of *Salmonella* bacterial culture on *Salmonella* Shigella Agar media.

**Figure-2:**
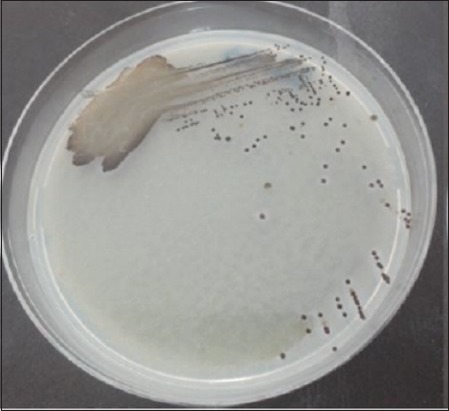
Result of *Salmonella* on Bismuth Sulfite Agar media.

**Figure-3:**
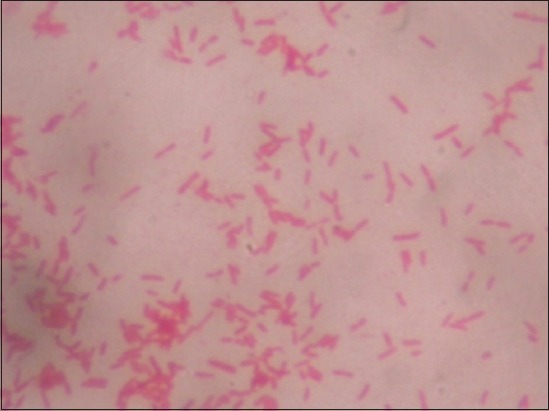
Microscopic examination result of *Salmonella* with negative Gram staining.

**Figure-4:**
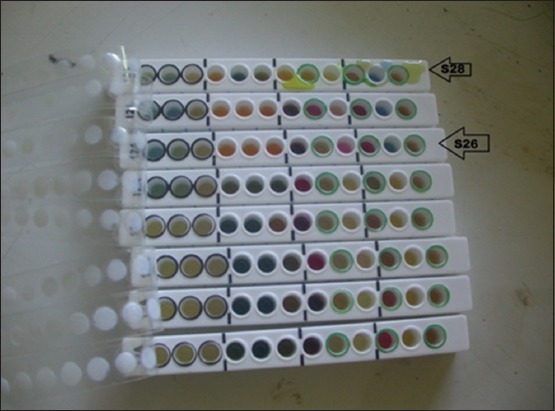
Positive *Salmonella* on biochemical test results using Microbact identification kits (arrows indicate positive *Salmonella*).

Testing on SSA media is done by macroscopic observation. On macroscopic examination, *Salmonella* growth produces transparent or colorless colonies with blackish color in the middle due to H_2_S gas formation (Figure-1). Macroscopic colony examination results on SSA media same on BSA media that showed 32 positive samples from 84 samples (38.09%) (Figure-2) and is shown in Table-1. These results were determined as *Salmonella* on Gram staining (Figure-3) and biochemical test (Figure-4).

**Table-1:** Results of *Salmonella* isolation and *invA* gene detection.

	Milkfish samples	*Salmonella*	*invA* gene
Total number	84	32	4

### Detection of encoding gene *Salmonella*

**Figure-5:**
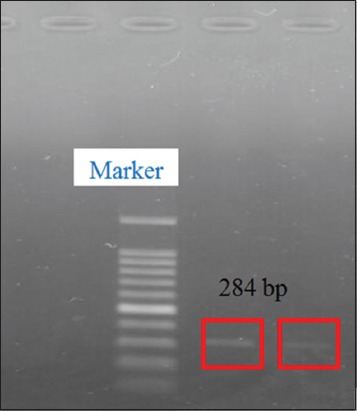
Results of detection of *inv* A gene encoding *Salmonella* on milkfish.

**Figure-6:**
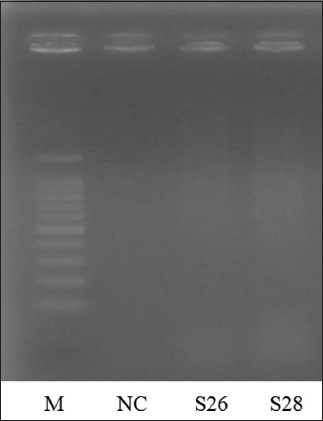
DNA visualization of *invA* gene *Salmonella* amplification products on a 1.5% agarose gel (tris-borate-ethylenediaminetetraacetic acid). Lane M=DNA marker 100 bp ladder, lane NC=*S. aureus* DNA as a negative control, lane S26; S28 positive *Salmonella* without positive result of *invA* gene *Salmonella*.

The results of detection of *Salmonella* invasive encoding genes performed using PCR tests shown in Figure-5, which shows positive *Salmonella* *invA* genes at 284 bp, and *Salmonella* which did not have *invA* genes (Figure-6).

*Salmonella* infection is a major bacterial disease in fishes, poultry, and humans, causing significant economic loss and illnesses [10,14,15]. Out of 84 samples, we isolated 32/84 (38.09%) of *Salmonella* by conventional culture method, and out of 32 positive *Salmonella*, we confirmed by PCR 4/32 (12.5%). Based on the results of PCR, there were 4 samples from 32 samples of *Salmonella* isolates which detected an *invA* gene with a length of 284 bp, while 28 samples did not show the target band. The absence of target bands in 28 samples showed that the strains of *Salmonella* bacteria obtained were not invasive or might also use other invasive mechanisms [16]. The use of certain PCR methods from *InvA* gene in most diagnostic and research laboratories is possible, and through molecular identification techniques of *Salmonella*, this method is the simplest and cheaper method [6].

At present, *Salmonella* is detected by standards bacteriological, biochemical, and serological techniques. These techniques are generally time-consuming, tedious, and expensive [17]. This research also corroborates some of the previous work carried out using the PCR method, in which non-typhoidal *Salmonella* was isolated from animals, food, and samples of human excrement in developing countries [18]. The observed prevalence of invasive *Salmonella* genes from poultry and poultry products is different from some other studies, which report a higher prevalence [6]. This variation may be due to differences in research methodology; gene-specific involvement, sample type, sample size and hygiene practices on the farm, and geographical location. *Salmonella* is known to infect animal products by various species; however, one serovar may be dominant in location for many years before being replaced by another. The most common and clinically significant serovar containing the *invA* virulence gene that causes salmonellosis globally is *S*. Typhimurium and *Salmonella enteritidis* [19].

Molecular methods are increasingly important in detecting and typing *Salmonella*. Tests are published with the aim of replacing traditional serotyping with direct detection of various genes [20]. PCR examination technique with the *invA* gene is very sensitive and specific in detecting *Salmonella*. Detection of *Salmonella*-specific by PCR with primers for *invA* gene is fast, sensitive, and specific to detect *Salmonella* in many clinical samples [21]. This research supports the ability of this specific primer set to confirm isolates as *Salmonella*. The *invA* gene contains sequences specific to the genus *Salmonella* and is considered the international standard for its characterization [16].

The *invA* gene is indeed targeted for the diagnosis of *Salmonella* organisms at the genus level [22]. *Salmonella*-specific primers’ ability to detect *Salmonella* species quickly and accurately in this study is mainly due to the primers sequence selected from the invasion of the *S*. Typhimurium gene as previously reported by Darwin and Miller [23]. This is also consistent with research conducted by Pererat and Murray [24] that the results of PCR on various serotypes of *Salmonella* showed positive results, while PCR results on non-*Salmonella* strains such as *E. coli*, *Klebsiella, Proteus*, and *Shigella* were negative for the *invA* gene.

First step in the intracellular pathogenicity cycle of *Salmonella* is the invasion of intestinal epithelial cells, and this step is controlled by the *invA* gene. The *invA* gene encodes proteins in bacterial cell membranes that are needed for invasion into host epithelial cells [23,25]. This gene is located in pathogenicity island I or referred to as *Salmonella* Pathogenicity Island (SPI); the DNA region is related to the pathogenicity of *Salmonella enterica* and is owned by all serotypes [26]. SPI serves to add complex virulence by bacteria to the infected host [24].

Many invasion genes encoded in SPI I and their expression are activated by the *Hil* A gene; transcription factors are also encoded in SPI I. In a study conducted by Murray and Lee [26] regarding the role of *Salmonella* invasion genes during infection in mice after intragastric inoculation, it was found that strains containing invasion genes that had mutations were found from intestinal tissue cells, and systemic tissue compared to strains that had not undergone previous mutation.

Galan *et al*. [27] also identified the *invA* gene as the first gene consisting of at least two additional invasion genes that are *Salmonella* to enter culture epithelial cells, but now there is an *invA* gene that has mutations. *S*. Typhimurium *invA* mutants are less capable of invading culture epithelial cells. *InvA* mutants do not alter the normal structure of the microvilli of epithelial cells and also do not cause changes in the distribution of actin microfilaments from cells. *InvA* mutants are significantly inhibited in their ability to enter cells, no emergence of invasive gene bands can also be caused by mutant genes that are unable to replicate on the intestinal mucosal surface, it can also be caused due to susceptibility to bactericidal host factors such as phagocytes. The invasion gene has been found in *Salmonella* with its ability to kill phagocytes *in vitro*.

With PCR, a single gene product can be strengthened in a single PCR reaction and in this study is shown as a rapid method for identifying one gene related to invasion virulence. Such early detection can have major benefits in public health, especially for rapid diagnosis and finding the ideal vaccine, epidemiological investigations, and prophylactic strategies for Salmonellosis in Sidoarjo, Indonesia. In summary, the findings from this study showed the distribution of *invA* gene (12.5%) in milkfish sources (Table-1). This requires planning and implementing control programs to prevent and control infections for spreading to public health.

## Conclusion

This study describes the prevalence of *Salmonella* contamination in the milkfish samples obtained from Sidoarjo wet fish market, Indonesia. The study found contamination of *Salmonella* in milkfish, indicating potential sources and routes of the *Salmonella* transmission in the fishery. Isolation of *Salmonella* carrying invasion gene (*InvA* gene) in this study could show poor sanitation of the environment in which milkfish are sold at wet fish market Sidoarjo, East Java Province, Indonesia.

## Authors’ Contributions

MHE supervised the project. SMY was a project leader and RPR carried out bacterial isolation and molecular work. FJW collected the samples. All authors contributed in the drafting and revision of the manuscript. All authors read and approved the final manuscript.
